# Limited Genomics Training Among Physicians Remains a Barrier to Genomics-Based Implementation of Precision Medicine

**DOI:** 10.3389/fmed.2022.757212

**Published:** 2022-03-18

**Authors:** Valerie M. Schaibley, Irma N. Ramos, Raymond L. Woosley, Steven Curry, Sean Hays, Kenneth S. Ramos

**Affiliations:** ^1^Center for Applied Genetics and Genomic Medicine, The University of Arizona Health Sciences, Tucson, AZ, United States; ^2^Genetic Counseling Graduate Program, University of Arizona College of Medicine – Tucson, Tucson, AZ, United States; ^3^Department of Cellular and Molecular Medicine, University of Arizona College of Medicine – Tucson, Tucson, AZ, United States; ^4^Department of Health Promotion Sciences, University of Arizona Mel and Enid Zuckerman College of Public Health, Tucson, AZ, United States; ^5^Division of Clinical Data Analytics and Decision Support, Department of Medicine, University of Arizona College of Medicine – Phoenix, Phoenix, AZ, United States; ^6^SciPinion, Bozeman, MT, United States; ^7^Institute of Biosciences and Technology, Texas A&M Health Science Center, Houston, TX, United States

**Keywords:** precision medicine, genetics, genomics, clinical care, training

## Abstract

The field of precision medicine has undergone significant growth over the past 10 years. Despite increasing applications of clinical genetic and genomic testing, studies consistently report limited knowledge of genetics and genomics among healthcare providers. This study explored barriers to the implementation of precision medicine by surveying physicians working in a large academic medical center. We assessed prior training in genetics, use of genetic testing in the clinic, desire for additional resources in genetics and genomic medicine and perceived barriers to successful integration of precision medicine. Only 20% of respondents reported moderate or extensive training in genetics. Physicians with limited or no training in genetics were less likely to have ordered a genetic test for any purpose. Furthermore, 41% of physicians responded that their lack of training identifying appropriate genetic tests and how to interpret genetic testing results was the most significant barrier to ordering genetic testing for their patients. These findings suggest that future efforts to realize the promise of precision medicine should focus on the integration of training programs for non-genetics trained healthcare providers.

## Introduction

Precision medicine is quickly expanding into mainstream clinical care. With the projected growth of precision medicine in the coming years, genetics and genomics will become an increasingly mainstream component of routine clinical care (1). As the taxonomy of disease is redefined on the basis of genetic and genomic insights, providers and healthcare systems will increasingly need to integrate genetic testing into patient care (2). Already, the rise in genetic testing has increased demand for clinical genetics services in the United States (3). This has collided with workforce shortages in clinical genetics, with many practices unable to take new patients and significant job vacancies for medical geneticists and genetic counselor positions across the country (3).

With increasing recognition of the link between chronic diseases and genomics and an inadequate number of healthcare providers (HCP) trained in genetics, the care of patients and families will fall to non-genetics trained providers. However, research has shown that many HCPs are not familiar with genetic testing procedures and test interpretation, and lack confidence in providing genetics-informed care and discussing genomics and genetics topics with their patients (4). Limited physician knowledge of genomics and genetic testing is repeatedly cited as a barrier for the implementation of precision medicine (5–8). Furthermore, lack of HCP knowledge of technological advances in the area of genetics has the potential to exacerbate health disparities in access to genetic testing among traditionally underserved populations (6).

In order to assess HCP knowledge and opinions on precision medicine and the integration of genomic medicine into their clinical practice, we surveyed HCPs working in a large academic medical center in central Arizona. The survey focused on the use of genetic testing in clinical practice, comfort using this type of data, and perceived barriers to expanding the use of clinical genetic testing.

## Materials and Methods

Survey questions were developed to evaluate physicians’ knowledge and opinions of precision medicine. Participants were asked 12 questions to assess their use of genetic testing and ability to capitalize on this data in the clinic (Table 1). The complete survey is provided as Supplementary Material. Participants were not required to complete all questions in the survey.

**TABLE 1:** Summary statistics for binary response questions.

Question	N “Yes” responses	Percentage “Yes” responses
Have you ever ordered a genetic-based test for diagnostic purposes?	33	52%
Have you ever ordered a genetic test for one of your patients?	36	56%
Do you feel you need help interpreting clinical genomic data if these data was made available to you?	55	86%
Do you think precision medicine will help define standards of care in medicine?	53	85%
Would you place a consult to a physician with expertise in genomic medicine if available at your institution?	55	86%
Would you like to attend trainings on precision medicine and genomics based testing for diagnostic purposes?	50	78%

A letter requesting participation in the survey was distributed to the entire physician staff at Banner - University Medical Center in Phoenix, Arizona (BUMCP). BUMCP is the primary academic medical center located in downtown Phoenix and is affiliated with the University of Arizona College of Medicine – Phoenix.

In compliance with institutional policy for human subjects research, the protocol was reviewed by the Banner Office for Human Research and The University of Arizona Institutional Review Board and deemed exempt from full review. All survey responses were anonymous. Sixty-four individuals completed the survey between October 30, 2017 and November 7, 2017 and the survey was closed on November 10, 2017. The survey was distributed, and responses were collected using a proprietary platform developed by SciPinion^1^.

**FIGURE 1:**
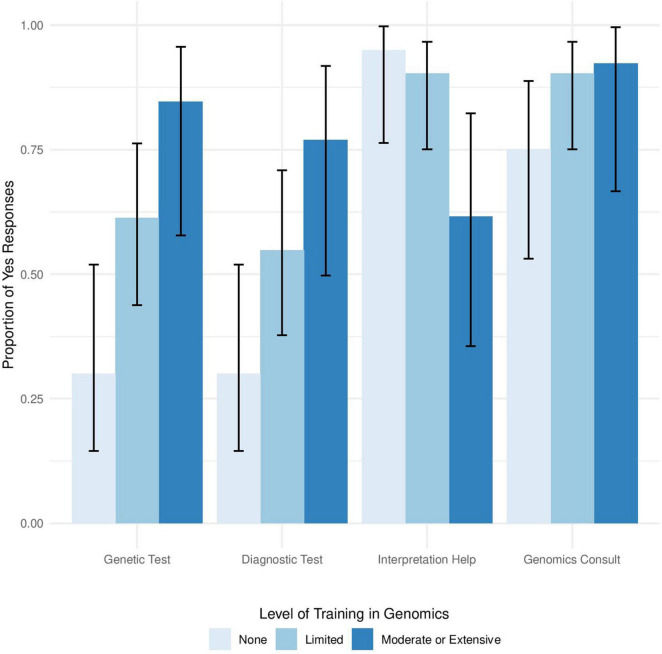
Level of genomics training influences use of genetics testing in clinical settings. Proportion of providers who responded Yes to the questions, “Have you ever ordered a genetic test for one of your patients?,” “Have you ever ordered a genetic-based test for diagnostic purposes?,” “Do you feel you need help interpreting clinical genomic data if these data was made available to you?,” and “Would you place a consult to a physician with expertise in genomic medicine if available at your institution” arranged from left to right stratified by self-reported level of training in genomics.

Survey results were analyzed using the Pearson’s chi-squared test for independence or a Chi-square goodness of fit test. Yates correction was used for all Chi-squared analyses to correct for the low sample size. The 95% confidence intervals shown in Figure 1 were calculated using the Wilson method. Statistical analysis was conducted in R version 3.5.1 and RStudio version 1.2.1335.

Responses to the single open-ended question in the survey (“What is your definition of precision medicine?”) were coded into five categories: (1) tailoring medical care to an individual, (2) using genetics to guide medical care (diagnosis, therapy, etc.), (3) understanding the combination of factors that influence health, like genetics, environment, and lifestyle, (4) basis for precision medicine is only based on genetic information, and (5) don’t know/no response. Reponses could fall into more than one category.

Responses to the question “What is your level of training in genomics?” that fell into the “Moderate” or “Extensive” categories were combined into a “Moderate or Extensive” category due to only a single respondent indicating they had extensive training in genomics. The response categories were not defined for this question, as we wanted respondents to self-assess their level of training and experience in genomics as “None,” “Limited,” “Moderate,” or “Extensive.”

## Results

### Study Summary and Description of Data

A 12-question survey was offered to providers working at a large academic medical center with practices in Phoenix, Arizona. The study was designed to assess the level of knowledge of precision medicine. An email invitation to the survey was distributed to physician providers at BUMCP using an internal listserv, with one reminder sent mid-way between October 30 and November 7, 2017. Sixty-four physicians completed the survey.

The majority of participants (86%) responded to all survey questions and most respondents were male (77%). Respondents completed medical training between 1971 and 2010. There was no significant difference between year of completion of medical training among respondents (Pearson’s χ^2^ = 4.75, *p* = 0.31). About half of respondents indicated that they had previously ordered a genetic test for a patient, with 52% of respondents reporting use of a genetic test for diagnosis in the past (Table 1). The survey showed that 86% of providers would welcome assistance with the interpretation of genetic testing results and that they would consult an expert in genomic medicine if the option was available (Table 1). Most providers (78%) indicated that they would attend training, if available, and that 85% believed precision medicine will define standards of care in the future (Table 1).

### Physician Definitions of Precision Medicine

Survey participants were asked to define precision medicine in their own words. A total of 59 responses to this question were provided. Three of the responses were specific, including a description of precision medicine as incorporating not just genetics, but other aspects of a patient’s health as well:

*“A treatment in medicine designed to take in account the patient’s illness in terms of variability in their lifestyle, genetic genome and environment. The treatment is aimed at defining particular group of patients with particular genetic genome that therapy maybe targeted for that group or prevention of illnesses associated that genome.”*

In contrast, about 10% of respondents replied to this question with, “I don’t know,” or “No idea,” suggesting that providers’ understanding of precision medicine varies widely (Table 2). Most respondents included a description of tailoring medical care to an individual, typically indicating that genetics was a primary driver of precision medicine (Table 2). Only 5% of respondents indicated that precision medicine encompasses more than just genetics (Table 2), suggesting that for these providers, genomics is the most significant element of precision-medicine based healthcare.

**TABLE 2:** Categorized responses to open ended question*.

Category	*N*	Percentage
Tailoring medical care to an individual	45	76.3%
Using genetics to guide medical care (diagnosis, therapy, etc.)	42	71.2%
Combination of factors that influence health (genetics, environment, and/or lifestyle)	3	5.1%
Basis for precision medicine is only genetic information	31	52.5%
Don’t know/no response	6	10.2%

*Responses to the question, “What is your definition of precision medicine?” were coded into five categories. Responses could fall into more than one category.

### Level of Training in Genomics Influences Use of Genomics in Clinical Settings

Chi-square goodness of fit analysis showed a significant difference between the level of training in genomics among respondents (χ^2^ = 30.125, *p* = 1.299e-6), with 80% of respondents reporting limited to no training in genomics. We found a statistically significant relationship between provider level of training in genetics and whether or not providers would order a genetic test (Pearson’s χ^2^ = 10.17, *p* = 0.006) or use a genetic test for diagnosis purposes (Pearson’s χ^2^ = 7.2, *p* = 0.027). Providers with Moderate or Extensive training in genetics more frequently ordered genetic tests than providers with limited training in genetics (Figure 1). Furthermore, providers with moderate or extensive training in genomics more frequently used genetic testing for diagnostic purposes than providers with limited or no advanced training in genomics (Figure 1). There was no significant relationship between respondents’ level of genetics training and year of completing medical training (Pearson’s χ^2^ = 8.5, *p* = 0.7483).

In addition to utilizing genetic testing in the clinic, level of training in genomics also influenced how providers would use that data in the clinic. There was a significant relationship between level of training in genomics and desire for assistance with interpretation of genetic test results (Figure 1, Pearson’s χ^2^ = 8.26, *p* = 0.016), with providers reporting less genomics training indicating that they would like help with genetic test interpretation. In contrast, no significant relationship was found between the level of training in genomics and the desire to consult an expert in genomic medicine (Figure 1, *p* = 0.3974), suggesting that providers generally agree that they would benefit from consulting with an expert in the field, regardless of level of training in genomics.

### Barriers to Implementation of Genetic Testing in the Clinic

Providers were asked to choose the most significant barrier for them when considering the option of ordering a genetic test for any given patient. The responses could be (1) availability of genetic tests, (2) personal training or knowledge of which genetic tests to order and how to integrate results from specific genetic tests, (3) medical guidelines, (4) cost, (5) lack of available therapies that are specific to genetic profiles, or (6) lack of confidence in therapy(ies) when they are available for specific genetic profiles.

**FIGURE 2:**
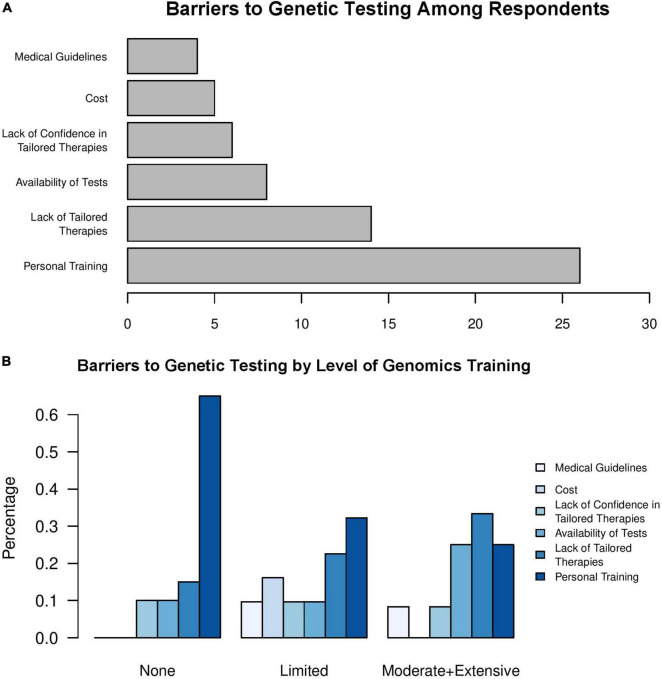
Lack of personal training in genomics is a barrier to clinical genetic testing. Physician’s responses to the question, “What is the major barrier for you to order a genetic test for a given patient?” among **(A)** all respondents and **(B)** stratified by level of training in genomics.

Chi-square goodness of fit analysis comparing the reported barriers to implementing genetic testing in clinic found a significant difference from a uniform distribution (Figure 2A, χ^2^ = 33.476, *p* = 3.027e-6). In our survey, 26 providers (41%) indicated that personal training on which genetic tests to order and how to interpret results from specific genetic tests was the most significant barrier to ordering a genetic test for a patient. As expected, we identified a relationship between the level of training in genetics and perceived barriers to testing (Figure 2B, Pearson’s χ^2^ = 6.9959, *p* = 0.03). For individuals with no training in genomics, lack of personal training was the most common response (Figure 2B), with over 60% of respondents who had no training in genomics indicating that this lack of training was the major barrier to ordering genetic tests for their patients. As respondents’ level of training in genomics increased from limited to moderate or extensive, lack of personal training in genomics was still a common barrier to genetic testing. However, these groups also reported that lack of tailored therapies or availability of testing were additional barriers that restricted the use of clinical genetic testing (Figure 2B).

## Discussion

Lack of provider knowledge in genetics has long been recognized as a barrier to the large-scale adoption of genetic testing into mainstream clinical care (5–8). However, despite a growing trend of increasing educational programs in genetics and genomics oriented toward non-genetics HCPs (9), recent studies suggest that many primary care physicians continue to be lacking in knowledge and confidence in clinical genetics and genomics (10, 11). In our study, participants cited that a lack of personal training was the most significant barrier to the use of genomics data in the clinic. Furthermore, we found that providers with higher levels of training were more likely to have ordered a genetic test, suggesting that additional training in genomics made them more comfortable with integration of genetics and genomics data into their practice. Together, these data support the claim that lack of training in genetics remains a significant obstacle in the expansion of genetics and genomics into clinical care. As such, training programs that teach fundamental concepts in genetic testing utilization may help non-genetics healthcare providers become more comfortable using this type of testing.

While our study found that roughly 50% of respondents have used some form of genetic testing in the clinic, 86% would like to have help interpreting these data. Furthermore, respondents in all groups wanted a genetics consulting service, even the single provider reporting “Extensive” training in genetics and genomics. Of note, the category with the most respondents who answered “No” to the question about genetics consultation was the group who indicated they had no advanced training in genetics (*n* = 5). This suggests that this group may not use genetic testing enough to warrant a consulting service, are unaware of the potential benefit of a genetics consult to help order and interpret genetic tests and genomics data, or feel that there is insufficient data to support the costs of genetic testing.

As genetics and genomics knowledge becomes commonplace, primary care practice will be heavily impacted by a massive inflow of genetic and genomic data. This will be especially exacerbated in medically underserved populations (6) and will only grow as consumer-initiated genetic testing expands (12), and return of genetic testing results in research programs grows (13, 14). Our findings suggest that creation of referral clinics within large healthcare settings may be an accelerator to the adoption of genetics and genomics data into clinical practice. However, genetics specialists are limited. While there has been recent growth in the genetic counseling profession, with the expansion of genetic counseling training programs in the United States (15), there will likely continue to be high demand for medical geneticists until gaps in training programs are addressed (3). Novel approaches to referral clinics, such as leveraging genetic counselors’ skills in providing information and interpretation of genetic testing to both patients and providers, while allowing medical geneticists to focus on complex diagnoses and management, could be a model to help address access to genetics specialty care while demand for medical geneticists continues.

Our study expands on existing literature showing that non-genetics trained healthcare providers are not comfortable implementing genetics into their clinical practice (4, 10, 11). Our study was unselected for physician specialty in order to broadly capture providers comfort and experience with genetic testing, outside of formal training and this may have led to discrepancies between individual responses. As such, future studies that examine the knowledge and comfort of using genetic testing among specialists and primary care providers would further clarify how well-equipped physicians in different areas of medicine are to incorporate genetic testing into their clinical practice. This could be further expanded by examining providers working in more diverse healthcare settings and geographic regions, where access to genetics specialists may be severely limited.

In our survey, most respondents indicated that precision medicine will define standards of care, with genetics having an increasingly prominent role in clinical practice. This calls for organized efforts by health care organizations to expand genetics and genomics education for both genetics and non-genetics providers to meet the future growth and demand for these types of services. Such expansion should include standardized interpretation resources, continuing education programs for providers and genomics consultation services.

## Data Availability Statement

The original contributions presented in the study are included in the article/Supplementary Material, further inquiries can be directed to the corresponding author.

## Ethics Statement

The studies involving human participants were reviewed and approved by the Banner Office for Human Research and The University of Arizona Institutional Review Board. Written informed consent for participation was not required for this study in accordance with the national legislation and the institutional requirements.

## Author Contributions

IR, SH, and KR were responsible for the conception and design of the work. VS and KR wrote the manuscript. All authors participated in drafting and revising the manuscript critically for intellectual content, approved of the final version to be published, and agreed to be accountable for all aspects of the work by ensuring that questions related to accuracy and integrity of any part of the work are appropriately investigated and resolved.

## Conflict of Interest

The authors declare that the research was conducted in the absence of any commercial or financial relationships that could be construed as a potential conflict of interest.

## Publisher’s Note

All claims expressed in this article are solely those of the authors and do not necessarily represent those of their affiliated organizations, or those of the publisher, the editors and the reviewers. Any product that may be evaluated in this article, or claim that may be made by its manufacturer, is not guaranteed or endorsed by the publisher.

## Footnotes

1
https://scipinion.com/
