# Supplementary material for: Limited Genomics Training Among Physicians Remains a Barrier to Genomics-Based Implementation of Precision Medicine
Source: Front Med (Lausanne). 2022 Mar 18;9:757212. doi: 10.3389/fmed.2022.757212 (PMC8971187; doi:10.3389/fmed.2022.757212)
Supplement: Supplementary file 1 [file Data_Sheet_1.docx]

**Banner-University Medical Center Phoenix Precision Medicine Survey**

1. When did you complete medical training? [select one]
   1. Pre 1950
   2. 1950-1960
   3. 1961-1970
   4. 1971-1980
   5. 1981-1990
   6. 1991-2000
   7. 2001-2010
   8. Post 2010
2. What is your gender?
   1. Male
   2. Female
   3. Not specified
3. What is your level of training in genomics? [list options]
   1. None
   2. Limited
   3. Moderate
   4. Extensive
4. Have you ever ordered a genetic-based test for diagnostic purposes?
   1. yes
   2. no
5. Have you ever ordered a genetic test for one of your patients?
   1. yes
   2. no
6. How often do you use genetic testing? [select one]
   1. Never
   2. Occasionally
   3. Frequently
   4. Daily basis
7. Do you feel you need help interpreting clinical genomic data if these data was made available to you?
   1. yes
   2. no
8. What is precision medicine? [text]
9. Do you think precision medicine will help define standards of care in medicine?
   1. yes
   2. no
10. Would you place a consult to a physician with expertise in genomic medicine if available at your institution?
    1. yes
    2. no
11. What is the major barrier for you to order a genetic test for a given patient?
    1. Availability of genetic tests
    2. Your personal training on knowledge of which genetic tests to order and how to interpret results from specific genetic tests
    3. Medical guidelines
    4. Cost
    5. Lack of available therapies that are specific to genetic profiles
    6. Lack of confidence in therapy(ies) when they are available for specific genetic profiles
12. Would you like to attend trainings on precision medicine and genomics based testing for diagnostic purposes?
    1. Yes
    2. No
